# “It isn't because they don't love their children”: social norms shaping young fathers' caregiving in Uganda

**DOI:** 10.3389/fsoc.2025.1568842

**Published:** 2025-11-26

**Authors:** Aloysious Nnyombi, Ramadhan Kirunda, Anslem Wandega, Moses Komagum, Deogratias Yiga, Kathryn M. Barker, Rebecka Lundgren

**Affiliations:** 1 Impact and Innovations Development Centre, Kampala, Uganda; 2 Department of Social Work and Social Administration, Makerere University, Kampala, Uganda; 3 Department of Social and Cultural Anthropology, University of Vienna, Vienna, Austria; 4 Center on Gender Equity and Health, Division of Global Public Health, University of California San Diego, La Jolla, CA, United States

**Keywords:** social norms, gender norms, caregiving, young fathers, early childhood development

## Abstract

This study investigates the influence of social norms on male caregiving in Uganda and considers the implications for tailoring and scaling the REAL Fathers mentoring program across six regions. By identifying key norms and reference groups, the findings inform strategies to enhance father engagement in early childhood development within culturally and socially relevant frameworks. This study employed an exploratory Participatory Learning and Action (PLA) approach, integrating in-depth interviews, focus group discussions, and community validation workshops to identify and prioritize social and gender norms across the study regions. We found that norms influence male engagement in care giving directly, by setting expectations for how men should behave with their wives and children. Norms also have a strong indirect influence through social expectations about what is appropriate for men and women related to power, decision-making and gender roles. Data shows that the norms and the reference groups that sustain them are consistent across cultural regions and the social sanctions that enforce the norms are largely intangible. We also found some exceptions to the norms. For norm shifting interventions to be effective, practitioners should be intentional in engaging reference groups and take advantage of exceptions to norms as leverage points for behavior change. Also, the different norm shifting strategies adopted should be able to address the complexity and interconnectedness of the norms.

## 1 Introduction

In Uganda, as in many low- and middle-income countries, prevailing gender norms contribute to the perpetuation of intimate partner violence (IPV) and limit fathers' involvement in early childhood development (ECD). Understanding how these norms operate—and who enforces them—is essential for designing effective, contextually grounded interventions that promote equitable and nurturing family relationships. The *Responsible, Engaged, and Loving Fathers (REAL Fathers)* program is an evidence-based, community-led, norm-shifting intervention that promotes positive parenting, gender equity, and non-violence among young fathers (ages 16–25) their partners, and communities. To inform the program's adaptation and scale up in six regions of Uganda, this study explored the social norms influencing IPV and fathers' engagement in early child development, as well as the key reference groups that uphold and enforce these norms. The research employed an exploratory Participatory Learning and Action (PLA) design and used a Social and Gender Norms Exploration (SGNE) approach guided by the Social Norms Exploration Tool (SNET) (Institute for Reproductive Health Georgetown University, 2020).

Gender and other social norms are unwritten rules about what is acceptable within a given group or community and can greatly influence an individual's choices and behaviors (Chung and Rimal, 2016). These implicit rules shape what people in a group believe is typical and appropriate behavior. Research suggests that both types of norms, descriptive (normal) and injunctive (approved) can affect behavior independently or together. Social expectations often play a powerful role in shaping family relationships, including how men interact with their wives and care for their children. They can perpetuate harmful practices and reinforce gender and other inequities, or uplift positive ones. Once established within a group, norms persist because people tend to conform or face social consequences for violating the expectations of their reference groups (Lapinski and Rimal, 2005). The strength of norms' influence is moderated by group identity and the rewards or sanctions associated with a behavior (Mackie et al., 2015).

Reference groups—those whose opinions and behaviors matter to individuals—create, transmit, and enforce social norms through rewards and sanctions (Bicchieri et al., 2014). They may include people whose approval individuals seek, whose advice they rely on, or whose behavior they emulate. Because reference groups strongly influence the adoption or reinforcement of social norms, behavior change interventions should carefully consider them when designing or adapting programs. It is also important to recognize that social norms are dynamic: they can shift over time even without formal intervention, and influence is bidirectional —individuals can affect their reference groups by introducing new behaviors or supportive messages (Cialdini et al., 1990). Finally, while norms are powerful, they are only one of many factors shaping behavior; economic conditions, poverty, migration, legal and policy frameworks, and service availability also play critical roles.

Building on current understanding of social norms and their influence on behavior, the REAL Fathers program catalyzes social norms change by engaging influential reference group members to stimulate reflection, build new skills, and model equitable, nonviolent behaviors. The program makes positive change visible within the community, publicly validates emerging norms, and diffuses new ideas through multiple, reinforcing channels. REAL Fathers employs four key program elements: father-to-father mentorship, group discussions with men and women, community billboards, and end-of-program community celebrations that publicly honor mentors and participating fathers. Together, these activities promote dialogue and recognition of equitable, nonviolent relationships, reinforcing community support for change (Kohli et al., 2025).

### 1.1 Social norms and father involvement in early childhood care, learning, and play

In the mind of the average Ugandan man, childcare roles are reserved for women. Men believe that they will not be taken seriously by their peers, wives, and broader community members if they are seen “wasting” time with children. For many men, fatherhood is limited to procreation and provision rather than a nurturing and playful father-child relationship. Research has been conducted to provide a deeper understanding of this dynamic. Watson et al. (2023) emphasize that culturally prescribed gender roles, which define men primarily as providers and advisors, often hinder men's desire to engage more deeply in areas such as child health and nutrition.

Nevertheless, in Uganda, as in other parts of the world, there is a growing trend of fathers participating in caregiving activities. Research has shown that fathers are more likely to contribute to child caregiving when the mother approves of their relationship with the child (Doherty et al., 1998), they maintain a healthy relationship with the mother (Varga et al., 2017), they are financially stable and identify as providers (Huffman et al., 2014; Högnäs and Williams, 2017) – and they feel confident in their parenting skills (Jones and Prinz, 2005).

Children need nurturing care to achieve optimal growth and development (Britto et al., 2017; Schwarzenberg and Georgieff, 2018). This requires responsive caregiving that starts in infancy through skin-to-skin contact, breastfeeding, and responding to baby faces or their talk by smiling, touching or talking back to them (Lagercrantz, 2016). It is also about ensuring security and safety, adequate nutrition, opportunities for early learning, and good health for young children (World Health Organization, 2018).

A father's participation in childcare influences many facets of childhood growth and development (Allen and Daly, 2007; Sarkadi et al., 2008). This has been associated with multiple positive physical outcomes. Male involvement during pregnancy reduces multiple medical risks at birth, including low birth weight, small for gestational age, preterm birth, and very preterm birth (Alio et al., 2010, 2011). Father involvement also moderates child sleep disturbances (Millikovsky-Ayalon et al., 2015).

Father participation also significantly improves children's cognitive functioning. Bronte-Tinkew et al. (2008) analysis of Early Childhood Longitudinal Study–Birth Cohort (9-month Father Study) data shows that cognitively stimulating activities, physical care, paternal warmth, and caregiving activities are associated with a lower likelihood of infant cognitive delay, particularly for male and infants with disabilities. Another study conducted among families from lower to middle-income backgrounds in Québec province Canada found that fathers' positive parental control predicted higher Performance Intelligence Quotient (IQ) (Pougnet et al., 2011).

The practice also positively influences positive socio-emotional and behavioral outcomes. A study by Kroll et al. (2016) showed that paternal positive parenting beliefs at age 9 months and increased frequency of creative play at 5 years were significantly associated with a lower risk of subsequent behavior problems in both boys and girls (Kroll et al., 2016). Another study showed that father involvement in outdoor leisure activities for children between 36 and 71 months of age predicts social competence and lower externalizing problems, particularly for boys (Torres et al., 2014).

An increasing body of evidence highlights the crucial role of father involvement in play, showing its significant impact on children's cognitive, emotional, social and physical development. Children receiving positive behaviors from their fathers during creative play demonstrate fewer behavior problems (Kroll et al., 2016), less aggression (DiLallo, 2003), better emotional regulation and higher receptive vocabulary (Cabrera et al., 2017). Those who undertake positive parenting behavior during free play support child-receptive language (Black et al., 1999), child-regulation compliance (Feldman and Klein, 2003) and child language development (Bornstein et al., 1992).

Despite the importance of fathers' involvement in early childhood care, learning and play, their direct participation in childcare remains limited. The International Men and Gender Equality Survey (IMAGES) in 32 countries shows that 61% of fathers have never bathed their child(ren), and 33% report having never played with their child(ren) (Equimundo, 2022). In Uganda, fathers primarily focus on teaching their children acceptable societal values while at the same time fulfilling their role as providers and protectors (AfriChild Centre, 2021).

Social norms play a significant role in limiting fathers' involvement in comprehensive childcare (van der Gaag et al., 2023). These norms often frame physical and emotional caregiving as inherently feminine, reinforcing the perception that men's roles in childcare are secondary and that women should bear the primary responsibility for raising children (Charmes, 2019). As a result, norms restrict the range of caregiving activities fathers consider, often excluding tasks like bathing, learning, and play from what they view as their responsibilities (Kalkstein et al., 2023).

## 2 Context and methods

### 2.1 The responsible, engaged, and loving fathers norm shifting intervention

The Responsible, Engaged, and Loving Fathers (REAL Fathers) program is an evidence-based, community-led norm-shifting intervention designed to reduce intimate partner violence (IPV) and violence against children while enhancing early childhood development and learning through play. The program specifically targets young fathers aged 18–25 with children aged 0–3 years, and, on a case-by-case basis, addresses the needs of younger fathers aged 16–17. Young fathers in this age group are at a pivotal stage of life, transitioning into new responsibilities. This makes them particularly open to adopting positive changes and new ways of life, which facilitates their transformation into REAL Fathers. Engaging these fathers meaningfully helps break the intergenerational cycle of violence and establishes a solid foundation for early childhood development and thriving family environments.

This study examined social and gender norms shaping IPV and fathers' engagement in early childhood development (ECD) for children under 3 years. It aimed to identify key reference groups influencing IPV and limiting father involvement, explore the norms that reinforce these behaviors, and co-design programmatic innovations within the REAL Fathers approach to address these norms. Findings were intended to inform strategies for promoting positive norms, reducing IPV, and enhancing fathers' participation in caregiving. Data collection and analysis for the social and gender norms exploration were conducted by the Impact and Innovations Development Centre (IIDC) under the REAL Fathers program.

### 2.2 Study setting

A total of six districts were purposively selected—one from each of the REAL Fathers Programme targeted regions of Ankole, Buganda, Bunyoro, Busoga, Teso and Lango. These included Rubirizi, Buikwe, Hoima, Kamuli, Busoga, Oyam and Amuria (Table 1). Thereafter, from each of the selected districts, sub-counties with the highest burden of violence against children were selected. Since the study was conducted in selected sub-counties within six of the 24 districts where the REAL Fathers Programme is being implemented, the study team conducted workshops to validate the findings by gathering input from the remaining 18 programme districts.

**Table 1:** Study sites.

**Region**	**Ankole**	**Buganda**	**Bunyoro**	**Busoga**	**Teso**	**Lango**
District	Rubirizi	Buikwe	Hoima	Kamuli	Amuria	Oyam
Sub-county	Katerera	Buikwe TC	Kyabigambire	Bulopa	Willa	Ngai
	Kicwamba	Nkokonjeru TC		Kagumba		

Primary data collection was conducted with 1,044 people including fathers aged 16–25 of children under three, fathers aged 16–25 with expectant wives, mothers to toddlers aged 0–3, expectant mothers, and reference groups (i.e., categories of people who influence the main population, advise them and can sanction or reward for both childcare). Validation workshops targeted persons from reference groups that were identified during the social norms' diagnosis study. Each validation workshop comprised 24 participants, with a total of 144 across all regions.

## 3 Methods

The study methods are adapted from the Social Norme Exploration Tool (SNET), a rapid, participatory approach to identify salient social norms and reference groups, inform the design of interventions to transform harmful norms and reinforce positive ones, and guide the development of monitoring and evaluation indicators (Institute for Reproductive Health Georgetown University, 2020). Qualitative data were collected through semi-structured interviews (IDIs) and focus group discussions (FGDs), using SNET participatory tools such as influence mapping, *My Social Network*, problem trees with the “Five Whys”, and vignettes. These tools enabled study participants to project their experiences and feelings indirectly, reducing social desirability bias by allowing them to discuss sensitive issues without speaking explicitly about themselves. Data related to social and gender norms surrounding intimate partner violence (IPV) and male caregiving were collaboratively analyzed and validated with community members, including identified reference group representatives and young fathers and mothers.

A total of 71 Focus Group Discussions (FGDs) were conducted across six regions. A purposive sampling strategy was employed to ensure the inclusion of participants with relevant experiences and perspectives on fatherhood and caregiving. Participants were drawn from four distinct sub-populations: (i) young fathers aged 16–25 years with children under the age of three; (ii) young fathers aged 16–25 years with expectant partners; (iii) expectant or pregnant wives of young fathers; and (iv) key reference group members, including mothers-in-law, community leaders, and male peers who shape social expectations and norms around fatherhood. Recruitment was carried out in collaboration with REAL Fathers partner organizations and local community leaders, who assisted in identifying and inviting eligible participants based on predefined inclusion criteria.

The FGD guides incorporated vignettes, short hypothetical scenarios depicting caregiving, IPV, and paternal involvement in childcare, to elicit participants' perceptions of descriptive and injunctive norms, anticipated social sanctions (approval or disapproval from others), sensitivity to sanctions (the extent to which they influence behavior), and perceived exceptions to norms (circumstances under which deviation is acceptable) (Bicchieri et al., 2014; Nguyen et al., 2020).

In addition, semi-structured interviews were conducted to identify reference group members who influence men's behaviors. Participants were asked to name individuals with whom they discuss issues related to male caregiving and to nature of their relationship with each person. They were then prompted to identify, among those listed, the key influencers and decision-makers who shape men's caregiving practices within their social networks.

Finally, validation workshops were conducted with participants drawn from reference groups identified through the social norms diagnostic study. The workshops incorporated presentations of preliminary findings, small group discussions, and plenary sessions designed to facilitate consensus building. All workshops were jointly facilitated by IIDC staff, REAL Fathers implementation partners, and government Community Development Officers. A total of six validation meetings were held, each bringing together representatives from four districts. Approximately 24 reference group members participated in each session. The workshops served to validate the identified norms and to identify additional norms that had not emerged during the initial data collection phase.

### 3.1 Data analysis

Data analysis began with the verbatim transcription of all audio-recorded data. Following transcription, the research team conducted repeated readings of the transcripts and coded the data according to the five components of the Social Norms Analysis Plot (SNAP)—a tool developed to guide systematic analysis of social norms data by identifying descriptive and injunctive norms, sanctions, sensitivity to sanctions, and exceptions (Nguyen et al., 2020). The coded data were then synthesized into descriptive and explanatory accounts to elucidate patterns and relationships.

### 3.2 Ethical considerations

All researchers received training in research ethics and successfully completed the Research Ethics and Compliance curriculum. In addition, they were trained on ethical engagement with participants, including procedures for safely referring individuals requesting assistance to appropriate local services and sources of psychosocial or health support. Study participation was voluntary, and ongoing informed consent was obtained from all study participants. Participants were discouraged from disclosing intimate personal details about their own experiences. To protect confidentiality and privacy, all data were anonymized by removing information, including names and location. The study protocol was reviewed and approved by the Makerere School of Social Sciences Research Ethics Committee (MAKSSREC) and the Uganda National Council of Science and Technology.

## 4 Results

This study identified a range of social norms that influence father's caregiving behavior. In this section, we discuss each norm, including mechanisms of enforcement and the circumstances under which exceptions occur. The norms identified pertain to household gender roles, men's time use, caregiving, discipline and play (Table 2). The results are presented together, as no meaningful differences were observed between men and women or between respondents from the six districts.

**Table 2:** List of social and gender norms.

**#**	**Norm label**	**Norm description**	**Dimension**	**Subregion**
1	Men and women are expected to play different roles	A man has specific roles, like providing for the family, while a woman cooks, washes clothes, fetches water, and digs, among others.	Gender	Ankole, Buganda, Busoga, Teso
2	Men should not participate in child caregiving roles	Young fathers are not expected to wash children's clothes and beddings, bathe, carry and feed babies/children. A man who does so is considered to have been bewitched by his wife and is disrespected	Social and gendered	Ankole, Buganda, Bunyoro, Lango, Teso
3	Real Men are not expected to return home early like chicken	Young fathers are expected to go to trading centers every evening by their peers	Gendered	Bunyoro, Busoga, Ankole
4	Children with disabilities should be kept hidden	It is typical of parents with children with disabilities to keep them hidden It is approved that children with learning difficulties are the responsibility of the mother	Disability	Ankole, Busoga
5	Parents are expected to use physical discipline	Parents correct their child's behavior through physical discipline	Age	Ankole, Buganda, Bunyoro

Analysis of the rapid interviews revealed 15 distinct reference groups, individuals who communicate and enforce social norms, including parents, paternal and maternal aunts and uncles, paternal grandmothers, brothers, health workers, peers, spouses, clan and religious leaders (Table 3). Notably, despite cultural and kinship differences across the six sub-regions, most reference groups were relevant across all areas Exceptions included boda boda cyclists, older brothers, and wives of elder brothers.

**Table 3:** Reference groups by region and category.

**No**	**Reference group**	**Sub-region**						**Cross-cultural influencer**
		**Ankole**	**Buganda**	**Bunyoro**	**Busoga**	**Lango**	**Teso**	
1	Mothers of young fathers	√	√	√	√	√	√	**Yes**
2	Fathers of young fathers	√	√	√	√	√	√	**Yes**
3	Maternal uncles	X	X	√	√	X	X	Yes
4	Paternal grandmothers	√	X	√	√	X	X	Yes
5	Older brothers	X	X	X	X	√	X	No
6	Health workers	√	√	√	√	√	√	**Yes**
7	Peers to young fathers	√	√	X	X	√	√	**Yes**
8	Spouses of young fathers	√	X	X	X	X	√	Yes
9	Maternal aunties	√	X	√	X	X	X	Yes
10	Cultural (Clan) leaders	√	√	√	X	X	√	**Yes**
11	Religious leaders	√	√	X	X	X	X	Yes
12	Brothers of young fathers	√	X	X	X	X	√	Yes
13	Sisters of young fathers	√	X	X	X	X	√	Yes
12	Boda boda cyclists	√	X	X	X	X	X	No
13	Mothers of the female spouses	√	√	X	X	√	X	**Yes**
14	Paternal aunties	X	√	√	X	X	X	Yes
15	Wives of elder brothers	X	X	X	X	X	√	No

### 4.1 Men and women are expected to complete different roles

Across the different regions, respondents and their reference group members believed that women hold the primary responsibility for caregiving, nurturing, and helping while men are responsible for providing for the family. In the Buganda, Busoga, and Teso regions, participants expressed the belief that meeting basic needs is solely the responsibility of men.

According to participants, such typical and appropriate behaviors are strengthened by religious scriptures on gender roles; for example, Proverbs 31: 27, “She watches over the affairs of her household and does not eat the bread of idleness”. Local proverbs also strengthen and enforce social expectations, for example, “Women who engage in heavy work develop sagging breasts.”

Study participants perceived that women and men would experience social punishments for playing roles not assigned to their gender. The most common forms of sanctions for men included being mocked, disrespected, discriminated against, not allowed to participate in community decisions and looked at as a lesser man bewitched by his wife. Female spouses of young men shared that they feel ashamed when they see their husbands doing house chores. Some reported that they intentionally burn their husbands if they find them cooking. Some young fathers do not conform to these expectations. However, they do not come out publicly to speak about their non-conformity out of fear of social punishment. Young men who follow the norm earn respect from community members and are applauded for being ‘real' men. The reference groups that reinforce this norm included young fathers and their wives, neighborhood men, clan leaders, wives of young fathers, In-laws, clan members, community leaders, and religious leaders.

Participants from the Ankole region shared that men from the Bahororo ethnic group could go against the norm if meat or fish sauce is to be prepared. This sauce is considered superior to others. Men are involved so that they have total control over how much sauce they eat.

### 4.2 Young fathers should not participate in child caregiving

Respondents and reference group members believed that men do not contribute to child caregiving and should not be responsible for child caregiving. In Ankole, female respondents perceived that childcare was their sole responsibility. In Buganda, reference group members expect men to commit less time to child care. In Lango, young fathers are not expected to care for and play with babies. In Busoga, reference groups do not expect men to care for mothers who have just given birth as they consider them “dirty.” By extension, young babies are deemed unmanageable because they are delicate like “eggs.” Reference group members also expect men not to care for children under 6 months of age to avoid contracting a skin disease when they touch the umbilical cord. In Bunyoro, young fathers are not expected to play with children.

Study participants perceived that men would experience social punishments when they contribute to child caregiving. The social punishments that they will likely experience include being seen as bewitched, controlled by the wife and lacking productive work. In Busoga, men who bathe children are perceived as persons who most likely wash their wives' knickers leading to questions about their masculinity. In Lango, men who bathe their children are perceived to be “women.” Also, young women do not allow their husbands to bathe their children for fear that they will be laughed at or gossiped about by other women. Young men who follow the norm earn respect from the community and are seen as real or strong men. The reference groups that reinforce this norm included peers of young fathers, local leaders, clan leaders, fathers to young fathers, religious leaders, paternal uncles, and elderly women.

Discussions revealed men could go against the norm when the spouse has passed away or in cases of divorce. The study identified some positive personal beliefs which when promoted can increase childcare by fathers. For example, some respondents believe that men who are involved in childcare are responsible people in the community with exceptional character. Others believe that a young father who spends time with the pregnant mother and children is a responsible and caring parent.

### 4.3 Real men should not return home early after work

In Teso, Ankole and Bunyoro respondents and their reference groups believed or expected that men do not return home early after work. In Teso, young fathers are expected to meet their peers in the trading centers every evening. Men shared that this is done to avoid perpetrating domestic violence. In Bunyoro, real men are not expected to return home early like chicken “abasaija tibataha kara nke'nkooko”. This is reinforced by the perception that men who stay close to their women live shorter lives. Spending a lot of time with your spouse is perceived to make it easy for women to bewitch men, especially if the man is having an extra-marital relationship. It also increases the possibility of the occurrence of events that bring about disputes. In Ankole, the spouse and the children must stay awake and only go to bed when the male spouse returns.

Most anticipated sanctions for deviating from the norm include being perceived as foolish and spoilt, not being in control of the home and probably having been bewitched by the spouse. Men are discriminated against in village meetings as their views are treated as those of a “child” or “woman.” They are also perceived by the community as idle, feminine and as men who lack better things to do.

Young men who follow the norm are respected, perceived as likely to lead long lives, praised by the community, and accepted within their peer groups. The reference groups that reinforce this norm include wives to young fathers, young fathers' peers, and elderly men.

### 4.4 Children with disabilities should be kept hidden and out of school

In Ankole and Busoga, most respondents and their reference groups believed or expected that children with disabilities should be kept hidden and out of school. Having a child with a disability is perceived to be shameful and abnormal. The implication is that families with a child with a disability face discrimination, as they are often viewed as bringing bad luck or a bad omen to the community. In Busoga, this norm shapes the expectation that children with learning difficulties belong to their mothers. The implication is that young fathers who abandon children with learning difficulties are not held accountable.

Most anticipated sanctions for deviating from the norm include young fathers being discriminated against if they prioritize the needs of a child living with a disability, men being encouraged to find another wife, or separation or divorce if the spouse prefers to prioritize the needs of the child with a disability. Young men who abide by this norm are praised by their relatives. The reference groups that reinforce this norm include young fathers, older men and women and local leaders.

### 4.5 Parents are expected to use physical discipline to correct their child's behavior

Most respondents and their reference groups believed and expected that parents should physically discipline their children. They shared that this is effective at shaping responsible and well-behaved members of the community. In Buganda, participants shared that they are instructed by the biblical scriptures to use harsh disciplining techniques, for example, Proverbs 13:24 “he who spares the rod hates his son, but he who loves him is diligent to discipline him”.

Study participants perceived that parents would experience social punishments when they deviate from the norm. These are gossiped about and are not consulted about issues to do with children. Also, parents who do not use harsh discipline techniques are looked at as not having control over their children. They are also considered non-exemplary to young parents. The parents who abide by the norm are praised for raising responsible children. The reference groups reinforcing this norm include young fathers, older men and women, local leaders, and religious leaders. In Bunyoro, participants shared that boys are not supposed to be beaten with a mingling stick—a traditional wooden stirring tool used to mix food. It is thought that this affects their reproductive system.

## 5 Discussion

We observe that gender and social norms related to household roles and caregiving participation confine young fathers‘ roles to economic provision while simultaneously assigning mothers the primary responsibility for caregiving. Similar findings have been reported in other literature, highlighting how social expectations emphasize men's roles as workers and providers (Flood and Howson, 2015), which limits fathers' identities to breadwinning and paid work (Doucet, 2020). For a young father in Uganda, this creates a binary opposition between caregiving and breadwinning, prompting them to prioritize paid work while side lining unpaid caregiving responsibilities. Comparable patterns have been observed worldwide. Research in European welfare states suggests that fatherhood ideals continue to prioritize full-time employment and economic support over caregiving (Martín-García et al., 2023). The State of the World's Fathers report, conducted across 17 countries, reveals that fathers are often perceived as primarily financial providers rather than emotional and physical caregivers (van der Gaag et al., 2023). Similarly, in sub-Saharan Africa, research highlights that cultural expectations and economic pressures position fathers as breadwinners (Van den Berg, 2015), while in Latin America, social norms portray fatherhood as complementary to motherhood rather than a shared responsibility (Franzoni, 2021). These findings suggest that the normative context described by this study is part of a broader global pattern linking fatherhood to provision.

In Uganda, this dichotomy is further reinforced by legal provisions that classify the inability to meet material needs as a form of child neglect.

The identified norms are consistent for both women and men and comparable across cultural regions. The cross-cultural relevance of these norms suggests that the approaches for shifting these norms can be similar. Consequently, the norms change initiatives implemented through the REAL Fathers program can be scaled across diverse cultural regions, providing significant opportunities for intercultural and cross regional learning. However, due to the unique manifestation of norms in specific areas, it is also essential to tailor behavior change efforts and resources for effective implementation of norms change activities at the community level.

The findings show notable similarities between the reference groups identified across the different cross-cultural regions. Since most of the reference group members belong to the local neighborhood, norms shifting efforts should focus on the close-knit community as a key leverage point during implementation.

The presence of nearly all key influencers within young fathers' immediate surroundings suggests that using a social network mapping approach and working through the community system offers a valuable opportunity to challenge norms and spread the transformation more effectively.

The norms identified in this study have varying levels of influence on the behavior of interest. Some have a direct influence on male caregiving. These explicitly suggest that young fathers should refrain from child caregiving. Consequently, this further entrenches the expectation that men should prioritize work over caregiving (Petts et al., 2018). There are norms that have a strong indirect influence on male caregiving through social expectations about what is appropriate for men and women related to power, decision-making and gender roles.

The social norm surrounding household gender roles operates as a “meta-norm” (Heise and Manji, 2016; Malhotra et al., 2019). As a higher-order norm, it delineates clear expectations for the division of labor and responsibilities between women and men. Our findings suggest that such a meta-norm significantly influences young fathers‘ behaviors in specific contexts, particularly regarding caregiving and family responsibilities. It shapes how young fathers interpret these situations, thereby perpetuating inequalities in caregiving. By emphasizing male involvement in the public sphere, this meta-norm creates sub-norms that restrict young fathers' engagement in child caregiving.

Certain norms create contradictory expectations. The norm surrounding child discipline offers young fathers opportunities to engage in caregiving while simultaneously imposing limits on how this involvement can occur. It endorses rules that promote harsh disciplinary techniques, which are associated with negative developmental outcomes (Satinsky et al., 2024). This contradiction signifies a missed opportunity, as effective and positive discipline is crucial for nurturing a child's self-discipline (Gfroerer et al., 2013), yet caregivers still don't appreciate the difference between disciplining and punishing. The REAL Father project works with families to understand the difference between the two.

The intricacies surrounding the social norms that underlie male caregiving require that practitioners adopt monitoring and adaptive management approaches which consider the uncertain and dynamic nature of complex situations with unclear cause-and-effect relationships. By adopting such approaches, social norm-shifting innovations should be able to strategically target points at which the different norms intersect. This supports the implementation of interventions that respond to the complex realities identified in this study.

The sanctions young fathers face for violating caregiving norms are severe and closely tied to their social identity and status. Notably, women also enforce these rules and may face repercussions if their husbands deviate from caregiving expectations. Young fathers are particularly sensitive to these sanctions, likely due to the fundamental human need for belonging, defined as “the subjective feeling of deep connection with social groups, physical places, and individual and collective experiences” (Allen et al., 2021:1). Since most sanctions are intangible and revolve around gaining or losing respect within the community, programs like REAL Fathers should co-design counter positive sanctions that resonate with individuals' sense of ego, social image, and community perception, ultimately fostering desired shifts in norms.

There were few exceptions to the norms—situations where it is considered acceptable to forego a social expectation (Cislaghi and Heise, 2017). These situations present key leverage points that can be used to pave the way for new, healthier norms in the community. For example, the exception that men can participate in child caregiving when the spouse has passed away or in cases of divorce demonstrates that men are capable and willing caregivers under certain conditions. Social norms shifting innovations can use these findings to identify positive deviants that can serve as change agents and challenge the perception that everyone conforms to the norm.

## 6 Study limitations

This study has a major focus on the social norms that influence young fathers' involvement in caregiving. From a socio-ecological perspective, a focus on norms is limiting as it does not provide an account of how social norms intersect with other factors to shape men's motivations and barriers to caregiving.

## 7 Conclusions

This article examines the social norms that shape father-to-child caregiving behaviors in Uganda. Our findings reveal that several norms influence fathers' involvement in childcare, learning, and play. These norms often reinforce the expectation that young fathers should prioritize their role as breadwinners. These norms, along with the key reference groups who reinforce the norms in communities, are largely consistent across different regions and population groups. In most cases, the associated rewards and sanctions for adhering to or violating these norms are intangible. However, some exceptions to these norms were observed.

From a data utilization perspective, we offer recommendations for how the REAL Fathers initiative and other norm-shifting interventions can apply insights from the social norms' exploration. This exploration highlighted key influence groups and exceptions to the norms. Norm-shifting interventions should leverage these as points for behavioral change. Since norms are embedded within these influence groups, targeting them can effectively support the process of shifting norms. The identified exceptions suggest opportunities to encourage critical reflection on current behaviors, thereby promoting the diffusion of a new normative framework where male involvement in caregiving is seen as obligatory, acceptable, and appropriate. Interventions should also consider the complexity and interconnectedness of norms that shape male caregiving behaviors, as these often overlap and influence young fathers‘ engagement in early childcare, learning, and play. The tools used to shift social norms must be capable of addressing these intersectional aspects to ensure a more comprehensive approach to changing behavior.

## Data Availability

The original contributions presented in the study are included in the article/supplementary material, further inquiries can be directed to the corresponding author.
